# Effect of Inter-Row Peanut Growing in the Vineyard on the Quality of ‘Cabernet Sauvignon’ Grape Fruits and Wines in Northwest China

**DOI:** 10.3390/foods11223730

**Published:** 2022-11-20

**Authors:** Jing Peng, Wei Wei, Haocheng Lu, Wu Chen, Shude Li, Chifang Cheng, Jun Wang, Changqing Duan, Fei He

**Affiliations:** 1 Center for Viticulture and Enology, College of Food Science and Nutritional Engineering, China Agricultural University, Beijing 100083, China; 2 Key Laboratory of Viticulture and Enology, Ministry of Agriculture and Rural Affairs, China Agricultural University, Beijing 100083, China; 3 CITIC Guoan Wine Co., Ltd., Urumqi 832200, China

**Keywords:** wine sensory, peanut, cover crop, aroma, phenolics

## Abstract

In order to solve the problem of premature grape ripening due to global warming, inter-row peanut growing in viticulture was applied. In this two-year (2018–2019) study, the peanut (*Arachis hypogaea* L.) was used to cover the ground between rows in the vineyards located in the semi-arid Northwest China, Xinjiang. The results showed that reflected solar radiation and temperature around the fruit zone with the peanuts growing were decreased. Compared with clean tillage, the grapes with covering peanuts had lower total soluble solids (TSS) and higher titratable acidity (TA) in the berries. Lower alcohol content and higher total acid (TA) was also found in their corresponding wines. Inter-row peanut growing treatment significantly decreased the contents of flavonols in the grapes and their wines in the two consecutive years, but no significant effect on flavanols was observed in the resulting wines. Norisoprenoids and esters in the grapes and the wines were increased with the peanut growing treatment, respectively. Additionally, compared to clean tillage, the peanut covering significantly improved the sensory value of the wines, especially the aroma complexity of the wines. This study helps us to better understand the feasibility of applying inter-row peanut growing in the viticulture of ground management in the semi-arid climate of Northwest China.

## 1. Introduction

Wine quality is influenced by many factors, of which vineyard management techniques are a key parameter. The different vineyard management techniques influence the vineyard climate, such as soil and microclimate [1]. The results of a multi-year study carried out in the vineyards of Bordeaux shows that climate has the greatest influence on the grape properties and the resulting wine quality [2].

In the different wine regions, there are sundry climatic conditions. The canopy microclimate of the vineyard is also important, as it affects the composition of grapes and wine [3,4,5]. The vineyard microclimate has become one of the main topics of modern research. In addition to cover crops, common vineyard measures that modify the vineyard microclimate include leaf removal, canopy shading and spur-pruning [6,7].

In sustainable practices implemented in the past, the use of covering crops remained controversial. However, it is true that the introduction of covering crops (weeds, legumes and their management) represents an ecological approach to increase the content of soil organic matter, which could promote the development of healthy and productive soils compared to traditional clean tillage [8,9]. Many vineyards use cover crops as a soil management strategy worldwide, and the result is that each species of cover crop has its own advantages and disadvantages [10], however many growers prefer to use legumes because of their ability in the fixation of atmospheric nitrogen, which can become available within a few months after planting cover crops [11].

In China, inter-row crop growing in viticulture has the advantages of being green, friendly, timesaving and labor-saving, and covering crops have gradually attracted more and more attention [12]. However, due to the vast territory and the great differences in the “terroir” of the different regions, the most suitable plant species for inter-row crop growing in viticulture in different regions is not the same. At present, by combining with the local climate and the production characteristics, different vineyard managers are gradually exploring the suitable species of plants that are used as covering crops in the vineyards worldwide. [13]. Usually, sensory evaluation of the resulting grapes and wines is used in these studies, the purpose of which is to clarify the influence of different technological treatments on their flavor, so that the experimental results have more practical significance for production [10]. At the same time, the wine sensory evaluation experiments can explore the sensory expression of the flavor substances with the perspective of molecular sensory science [12].

Sensory evaluation of wine is especially beneficial to the development of new wine products [14]. In the process of wine production and commercialization, sensory evaluation is helpful for the identification of the quality of wines. Winemakers need to understand the current situation and the developing changes and take measures to resolve the defects of the wines through wine sensory evaluation [15]. This wine sensory evaluation experiment applies the wines as the experimental material and adapts the method of panel training and evaluation to measure the appearance, aroma and taste of the wines, so as to provide a theoretical basis for accurately locating the sensory qualities of the wines, and further improving their qualities. Lan et al. used a related method, which also combined solid-phase extraction (SPE) to investigate the difference of the aroma profiles in ‘Petit Manseng’ wines among three wine regions in China, and the result showed that the method had certain guiding significance for wine quality identification [16].

To the best of our knowledge, there were only a few studies in Northwest China on the use of covering crops to determine the quality of grapes and wine, and there are no studies on inter-row peanuts in vineyards, especially those based on sensory evaluation [17,18,19]. In this study, the results show that covering peanuts could improve the aroma and taste expression in wines, offering great potential for producing high quality wines in this region, compared to clean tillage. In addition, the harvested peanuts could provide additional income for the grape growers.

## 2. Materials and Methods

### 2.1. Experimental Site and Design

In 2018 and 2019, when this experiment was conducted, the two consecutive years were normal years in Xinjiang according to local weather reports. We chose the same experimental vineyard as Peng et al. [19], which is located in Manas County, Xinjiang Uygur Autonomous Region, at the Northern Foot of the Tianshan Mountains. The variety used in the experiment was ‘Cabernet Sauvignon’ (*V. vinifera* L. cv.) vine planted in 2002, which was the own-rooted cultivar. The distance between rows and vines was 2.8 × 1.0 m. All vines in the experiment used a modified VSP (vertical shoot-positioned spur-pruned cordon system) [20] with a canopy height of 1.2 m and a width of 0.8 m. The cords were 0.6 m above the ground.

We monitored the grape development process and collected samples of grapes at five developmental stages (pea size, 5% veraison, 100% veraison, 10 days after 100% veraison and the commercial harvest stage (TSS ≈ 24°Brix)). In each block, three adjacent experimental rows were considered replicates. Three replicates were used with 20 vines of similar growth vigor per replicate. The 500 berries of each replicate were randomly sampled at each developmental stage from different parts of the cluster. After sampling, 100 berries were randomly selected to measure the physicochemical parameters and the rest were immediately frozen in liquid nitrogen and stored at −80 °C for the subsequent analysis of flavonoids and aromatic compounds.

The treatments were:

(1) Control: clean tillage between rows (CK);

(2) Treatment: peanuts (*Arachis hypogaea* L.) were grown between rows in the vineyards (CH).

In field management, peanuts were planted manually in spring and harvested in September. Clean tillage was used for control and weeds between the rows were removed regularly. Throughout the grape development, there was no inter-row irrigation in either treatment or control.

### 2.2. Climate and Microclimate Data Observation

At the experimental site, we obtained meteorological data from the China Meteorological Data Service Centre (http://cdc.cma.gov.cn/, 4 September 2022), and the meteorological data included average monthly temperature and sunshine duration during the grape growing season (from April to October) of 2018 and 2019. We installed microclimate stations (Hobo^®^ micro station, Onset Corporation, Bourne, MA, USA) in the fruit-zone of clean tillage and covering peanuts. The temperature and photosynthetically active radiation (PAR) were recorded every 5 min on a sunny day during the post-veraison and pre-harvest periods [19].

In 2019, one week before harvest, the main leaf area and the lateral leaf area of ten shoots per replicate was measured using a portable leaf area meter (Yaxin-1242, Beijing, China). The length and diameter (the third internode from the base) of ten shoots per replicate were measured. At harvest, in each replication, we calculated the weight of ten randomly selected bunches, the seeds from each berry and skin weight from each berry, and counted the number of bunches on ten vines, the seed number of each berry and the berry number from each bunch. At harvest, the grape yield was monitored by weighing the cluster weight of the vines. During winter pruning, the canes were pruned and weighed on five vines per replicate [19].

### 2.3. Analysis of Berry and Wine Physiochemical Composition

In terms of the physiochemical composition in grape berries and wine, we weighed one hundred berries. We detected the total soluble solids (TSS), the titratable acidity (TA) and the value of pH from the grapes by pressing them by hand. The TSS in grapes was measured using the PAL-1 digital handheld refractometer (Atago, Tokyo, Japan). The TA was analyzed by titration with NaOH (0.05 M) to a final pH of 8.2 and expressed as tartaric acid equivalents according to the National Standard of the People’s Republic of China [19,21]. The value of pH was measured with the Mettler LE438 pH meter (Mettler, Toledo, Switzerland).

In wines, the value of pH was determined with a pH meter (Sartorius PB-10, Göttingen, Germany). The total acidity (TA) of the wine was analysed in the same way as the titratable acidity of grapes. Prior to analysis, the carbon dioxide was removed with an exhaust device. According to OIV, we determined the residual sugar, volatile acidity and ethanol content of the wine [22]. As described in Ayala et al. [23], we used the standard CIELAB formulae to determine the wine colour parameters: lightness (*L*), red-green colour contribution (*a*), yellow-blue colour contribution (*b*), chroma (*C*) and angular hue (*H*) [19].

### 2.4. Extraction of Flavonoid Compounds in Berry Skins and Seeds

The berry peel and seed were manually shelled and selected. Then, the peel and seed were pulverised separately in the frozen state under the protection of liquid nitrogen and then dried at −40 °C under vacuum. The dried skin powder was used to extract anthocyanins, flavonols and flavan-3-ols. Dried seed powder was used to extract flavan-3-ols [19].

The flavonols and anthocyanins were extracted according to the procedure reported by Downey et al. [24] and He et al. [25]. Dried skin powder (0.100 g) was macerated and sonicated in 50% (*v*/*v*) methanol in water (1.0 mL) for 20 min. Extraction was performed by centrifugation for 10 min at 12,000 rpm. The supernatant was collected, and the residue was then extracted twice [19]. The flavan-3-ol was extracted according to Liang et al. [26] and Peng et al. [19]. To determine the concentrations of the different flavan-3-ol units, grape sample powder (0.10 g) was mixed with 1 mL of phloroglucinol buffer (0.5% ascorbate, 300 mmol/L HCl and 50 g/L phloroglucinol in methanol), incubated at 50 °C for 20 min, neutralized with 1 mL sodium acetate (200 mmol/L, pH 7.5) and finally centrifuged at 8000 rpm for 15 min. This procedure was repeated three times and the supernatants were pooled [19]. To prepare free flavan-3-ol monomers, 0.1 g of the dried sample powder was extracted in 1 mL of 70% acetone with 0.5% ascorbate, mixed, centrifuged and repeated twice. Then 400 µL of the combined supernatants was dried rapidly with a dry stream of nitrogen at 30 °C [19]. The dried samples were dissolved in 200 µL methanol acidified with 1% (*v*/*v*) HCl and neutralized with 200 µL aqueous sodium acetate (200 mM) [19,27].

### 2.5. HPLC-MS Analysis of Phenolic Compounds in Berries and Wines

We used high-performance liquid chromatography/triple-quadrupole tandem mass spectrometry (HPLC-QqQ-MS/MS) to determin the phenolic compounds in the berries and wine. An Agilent 1200 series HPLC with a Poroshell 120 EC-C18 column (150 × 2.1 mm, 2.7 μm) coupled to an Agilent 6410 triple-quadrupole (QqQ) instrument was used. According to the method described by Li et al. [28], we analysed the anthocyanins and non-anthocyanic phenolics. The free flavan-3-ols and proanthocyanidins were analysed according to the method described by Sun et al. [29]. The fresh berry weight (FW) in grapes, the concentrations of phenolic compounds were expressed as mg/kg and as mg/L in wines.

### 2.6. Extraction of Berry Aroma Compounds

Free and bound aroma compounds were extracted according to the method of Lan et al. (2016) [30]. For each replicate, 80 g de-seeded berries was ground with 1 g polyvinylpolypyrrolidone and 0.5 g D-gluconic acid lactone in liquid nitrogen, then was macerated at 4 °C for 4 h and centrifuged to obtain clear must. A quantity of 5 mL grape must was added in a 20 mL vial with 1 g NaCl and 10 µL 4-methyl-2-pentanol (internal standard). Bound aroma compounds were isolated by using Cleanert PEP-SPE resins, and enzymatic hydrolysis of glycosidic precursors was conducted at 40 °C for 16 h by adding 100 µL AR 2000 (Rapidase, 100 g/L). Samples were placed in a CTC-Combi PAL autosampler (CTC Analytics, Zwingen, Switzerland) equipped with a 2 cm DVB/CAR/PDMS 50/30 µm SPME fiber (Supelco, Bellefonete, PA, USA) and agitated at 500 rpm for 30 min at 40 °C. The SPME fibre was then inserted into the headspace to absorb aroma compounds at 40 °C for 30 min and was instantly desorbed into the GC injector to desorb aroma compounds.

### 2.7. GC-MS Analysis of Aroma Compounds in Grapes and Wines

Aroma compounds from grape and wine samples were extracted by headspace solid-phase microextraction (HS-SPME) and analysed by gas chromatography-mass spectrometry (GC-MS) as described by Wen et al. [31]. Agilent 6890 GC coupled with Agilent 5973C MS. GC was equipped with an HP-INNOW AX capillary column (60 m × 0.25 mm, 0.25 µm, J and W Scientific, Folsom, CA, USA) and used to separate volatile compounds. Qualitative and quantitative methods were used as described by Wang et al. (2019) [32]. The concentrations of volatile compounds were expressed as μg/L in wines and μg/kg of fresh berry weight in grapes.

### 2.8. Small-Scale Fermentation

For each replicate, the grapes were hand-picked from 20 vines and immediately brought to the laboratory. We applied the same methods of small-scale fermentation as Peng et al. [19], including alcoholic fermentation, malolactic fermentation, filtered, bottled and stored.

### 2.9. Sensory Evaluation

We used the method of panel training and evaluating to measure the appearance, aroma and taste of the wines, so as to provide a theoretical basis for accurately locating the sensory qualities of the wines, and further improving their qualities. The valuation consisted of describing the aspects of visual, aroma, taste and harmony found in the wine samples, which accounted for scores of 10, 30, 50 and 10, respectively [19,33].

### 2.10. Statistical Analysis

SPSS version 22.0 (SPSS Corporation, Chicago, IL, USA) was used for all significance analyses at *p* < 0.05 (Duncan’s multiple range test or t-test). The figures were drawn by using the Origin 2021b software (OriginLab, Northampton, MA, USA), Simca 14.1 (Umetrics, Malmö, Sweden) and GraphPad Prism 8.0.2. (GraphPad Software, San Diego, CA, USA).

## 3. Results and Discussion

### 3.1. Vintage Climatic Characteristics and the Influence of Treatments on Grapevine Microclimate

We have reported the mesoclimatic climatic conditions of the vineyard in 2018 and 2019 [19]. As shown in Table S1, according to the growing season temperature (GST, 19.6–20.4 °C), the experiment site was classified as a ‘Hot region’ [34]. We also calculated the value of heliothermal index (HI, 2741–2966 °C) and the growing degree days (GDD, 2011–2017 °C); the results show that the experiment site is in a ‘warm region’ [35,36]. These bioclimatic indices suggest that the climate of the experiment site could be described as dry hot, which is consistent with results of Peng et al. [19]. As shown in Table S2, the vintage 2018 was characterized as a cooler vintage with lower MesoT_v and longer lower temperature duration (DH10) compared to 2019, and the grapes in the year of 2018 had a later verison period and harvest period. Vintage 2019 had lower solar radiation and sunlight duration, but longer high temperature duration (DH 30) of the vineyard relative to 2018.

As shown in Table S3, covering peanuts decreased the average daily soil water content (SoilW), fruit-zone average daily temperature (MicroT), average daily photosynthetically active radiation (MicroPAR), and had higher DH10 and less high DH30. As we expected, covering crops decreased the DH30 and the MicroT while increasing the DH10. The peanut plants could absorb the heat and the water, which consequently resulted in decreased soil water content and fruit-zone average daily temperature, pro-shorted fruit-zone high temperature duration and more low temperature duration, and the result is consistent with the results of Peng et al. [19].

### 3.2. Effect of Covering Peanuts on the Grape Vegetative Parameters

Decreases in the leaf area, the leaf area/yield and the berry number were observed in the treatment of covering peanuts in 2019 (Table S4). Covering peanuts resulted in a 36.8% reduction in the total leaf area, a 9.6% reduction in the leaf area/yield and a 6.8% reduction in the berry number. Furthermore, compared to the clean tillage, covering peanuts also decreased the main shoot leaf area and the lateral shoot leaf area by 24.5% and 45.6%, respectively. Covering peanuts did not significantly affect the pruning weights and yields. Therefore, covering peanuts significantly decreased the value of leaf area/yield. The authors speculate the reasons might be the competition for nutrients between the peanuts and vines in the rows. This indicates that covering peanuts decreases the photosynthesis capacity, which results in a reduction in biomass. In addition, this study has results similar to Peng et al. [19] in average shoot length, third internode diameter, seed number, seed weight, skin weight and berry size.

In this study, the results of grape vegetative parameters are similar to results by Peng et al. [19], who studied the influence on quality of grapes and wine by covering purslane. Compared to the results with Peng et al. (2022) [19], covering peanuts and covering purslane both significantly decreased the leaf area and leaf area/yield. In terms of the leaf area, in theory, covering peanuts would increase exposure in the fruiting zone, but the results of this study showed that covering peanuts reduced the PAR. Peng et al. [19] explained that cover crops decrease the reflected light, which reduces the PAR. As for the leaf area/yield, covering peanuts and covering purslane did not significantly affect the yield of the grapes, but decreased the leaf area, thus decreasing the leaf area/yield.

### 3.3. Grape Physiochemical Parameters

**Figure 1:**
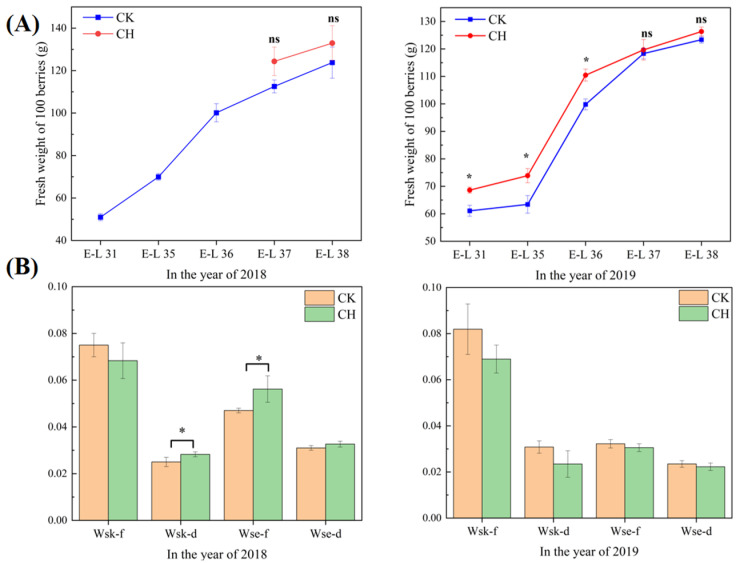
Effects of covering peanuts on berry weight in 2018 and 2019 (**A**), and skin weight and seed weight in 2018 and 2019 (**B**). Wsk-f, Skin fresh weight (g/berry); Wsk-d, Skin dry weight (g/berry); Wse-f, Seed fresh weight (g/berry); Wse-d, Seed dry weight (g/berry). Data are expressed as mean ± standard error (*n* = 3). Different letters with each stage indicate the significant differences based on Duncan’s test at *p* < 0.05, and ns indicates no significant differences between treatments. CK: clean tillage; CH: covering peanuts.

**Figure 2:**
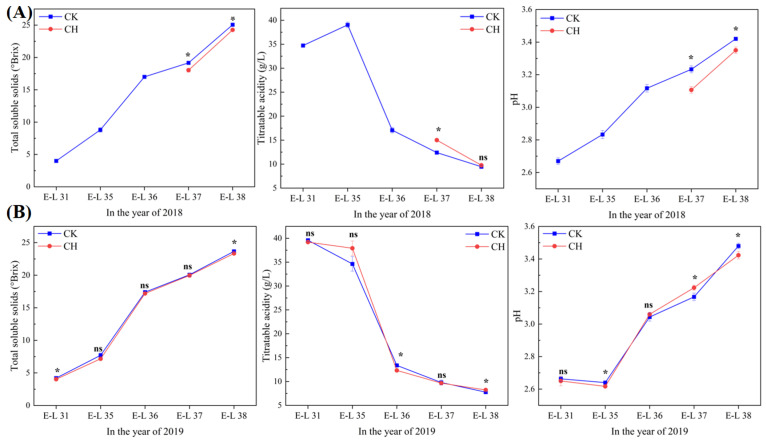
Effects of covering peanuts on the total soluble solids, titratable acidity and pH in 2018 (**A**), and in 2019 (**B**). Data are expressed as mean ± standard error (*n* = 3). Different letters with each stage indicate the significant differences based on Duncan’s test at *p* < 0.05, and ns indicates no significant differences between treatments. CK: clean tillage; CH: covering peanuts.

As shown in Figure 1 and Figure 2, there were significant differences on the berry total soluble solids (TSS), the titratable acidity (TA) and the pH value between treatments, especially at harvest. However, covering peanuts did not affect the berry weight and the skin weight at harvest. Covering peanuts decreased the TSS at E-L 37 and E-L 38 stages in 2018, and at E-L 38 stage in 2019. In addition, covering peanuts increased the TA and decreased the pH value at E-L 38 stage in 2019 (Figure 2). This indicates that covering peanuts could delay the accumulation of the soluble solids in wine grape berries.

Our results showed that covering peanuts was sufficient to affect the ripening process in the dry hot regions with strong light. Several studies reported effects on grape berry physiochemical parameters by covering crops, including *Portulaca oleracea* L. and *Arachis hypogaea* L. [8,19,37,38,39,40,41,42]. Those results indicated that the effects of cover crops on the berry composition might be related to the cultivation methods, climatic conditions and the change of microclimate, which should be considered before applying ground management of cover crops [39,43,44]. Compared to the results of Peng et al. (2022) [19], covering peanuts and covering purslane both significantly reduced the value of pH of grape berries (E-L 38). The authors speculate the reason is that covering peanuts and covering purslane delayed the accumulation of sugar in grapes and slowed down the grape-developing process.

### 3.4. Influence of Covering Peanuts on the Aroma Compounds and the Flavonoids in Grapes

In this study, we summed up the concentrations of aroma compounds in free form and bound form in the grapes berries, and investigated the influence of covering peanuts on the grape aroma compounds. There were a total of ninety aroma compounds in grape berries, including free form and bound form in the two years. As shown in Table S5, the aroma compounds fell into the following groups: norisoprenoids, C_6_/C_9_ compounds, benzenes, terpenoids, fatty acids, alcohols, aldehydes and ketones and esters.

**Figure 3:**
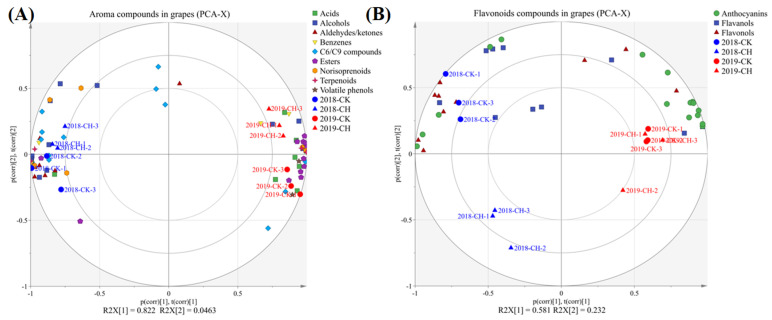
PCA based on aroma and flavonoids compounds concentrations separating grape samples according to vintage and treatment. (**A**) correlation loading plot in aroma compounds of grape berries, (**B**) correlation loading plot in flavonoids in grape berries.

**Figure 4:**
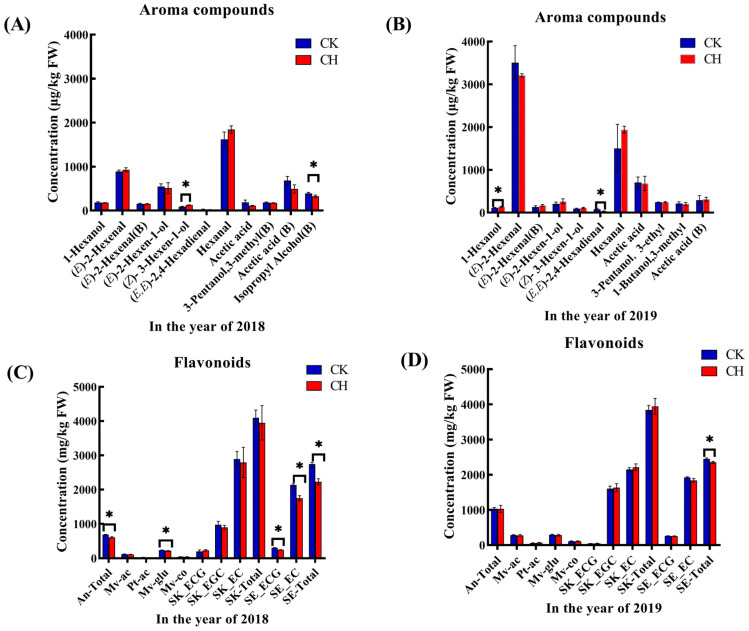
Effects of covering peanuts on the concentrations of the marker aroma compounds and flavonoids in the mature grapes in 2018 and 2019. The marker aroma compounds in 2018 (**A**) and 2019 (**B**); marker flavonoids in 2018 (**C**) and 2019 (**D**). Data in bar plots are expressed as mean ± standard error (n = 3). * indicates significant differences based on Duncan’s test at *p* < 0.05.

We used principal component analysis (PCA) to identify the variations between different treatments and vintages based on the berries’ aroma compounds, as shown in Figure 3A. The PC 1 and PC 2 explained 82.2% and 5.0% of the total variance, respectively, and the different treatments and vintages were separated from each other clearly. The loading plot showed that there were higher concentrations and proportions of the C_6_/C_9_ compounds and alcohols, as well as the norisoprenoids than those of 2019. The grapes with covering peanuts treatment were characterized by higher concentrations and proportions of the norisoprenoids and the C_6_/C_9_ compounds and lower concentrations and proportions of the acids and the aldehydes than those of the clean tillage. However, there was no significant difference in β-damascenone in grape berries by covering peanuts and clean tillage, and the main different compound was 6-methyl-5-Hepten-2-one. Compared to clean tillage, covering peanuts increased the concentration of 6-methyl- 5-Hepten-2-one, and further increased the concentration of norisoprenoids, as shown in Table S5. The primary biomarker aroma compounds identified by the OPLS-DA model are shown in Figure 4A,B, which were recognized as C_6_/C_9_ compounds, fatty acids and alcohols in 2018 and 2019. In 2018, covering peanuts increased the concentration of (*Z*)-3-hexen-1-ol and decreased the concentration of isopropyl alcohol. In 2019, covering peanuts increased the concentration of 1-hexanol and decreased the concentration of (*E,E*)-2,4-hexadienal.

As shown in Table S6, we report the concentrations and proportions of the anthocyanins, flavonols and flavanols in grapes berries. Since there was a difference of flavonoids in grape berries by covering peanuts and clean tillage, we built reliable PCA models from comparisons of clean tillage and covering peanuts, as shown in Figure 3B. Based on the grapes berries’ flavonoids, the PC 1 and PC 2 separated the vintages and treatments from each other, which explained 58.1% and 23.2% of the total variance, respectively. As shown in the loading plot, the result showed that there were higher concentrations and proportions of the flavanals and lower concentrations and proportions of the anthocyanins in 2018. The grapes with covering peanuts were characterized by higher concentrations and proportions of the anthocyanins, flavonols and flavanols than those of the clean tillage. Several studies shown that the influence of photosynthetically active radiation on the anthocyanins’ concentrations was related to the fruit-zone light exposure [45,46]. Compared to the results of Peng et al. (2022) [19], the effect on the anthocyanins, flavonols and flavanols by covering peanuts and covering purslane was inconsistent. Covering purslane significantly increased the concentration of the flavonols, but did not have a significant influence on the anthocyanins and flavanols. Covering peanuts reduced the concentrations of total anthocyanins and the total proanthocynidins in seeds. The author speculates the reason is that covering peanuts have the ability to fix the nitrogen in the soil compared to covering purslane.

The primary biomarker flavonoids identified by the OPLS-DA model is shown in Figure 4C,D. Covering peanuts decreased the total concentration of the anthocyanins and flavanols, the concentration of malvidin-3-*O*-glucoside, (-)-epicatechin (EC) and (-)-epicatechin-3-*O*-galate (ECG) in the seeds. There were no significant differences in the concentrations of flavonols between clean tillage and covering peanuts in grape berry.

### 3.5. The Correlations between the Climatic Parameters and the Flavor Compounds

**Figure 5:**
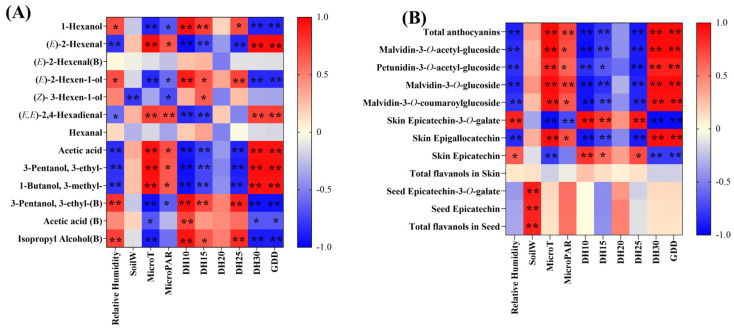
Pearson correlation analysis revealing the relationships between the climate indicators and the marker aroma compounds (**A**), and the marker flavonoids compounds (**B**). Different cell colours in the heatmaps represent a positive (red) or negative (blue) correlation. ** represents significant correlations at *p* < 0.01 and * represents significant correlations at *p* < 0.05.

As shown in Figure 5, we pooled the concentration of the marker aroma compounds and the marker flavonoids compounds, the mesoclimatic and microclimatic indices of the two vintages, and conducted a partial least square regression (PLSR) analysis, in order to explore the causes of differences in the concentrations of the aroma compounds and flavonoids compounds between vintages and between treatments [37]. In terms of the aroma compounds, the concentration of hexanal and bound (*E*)-2-hexenal did not have correlations with the mesoclimatic and microclimatic indices. The concentration of acetic acid, 3-pentanol, 3-ethyl, and 1-butanol, 3-methyl had the same correlations with the climatic parameters. The concentration of 1-hexanol, (*E*)-2-hexanal, bound 3-pentanol, 3-ethyl and bound isopropyl alcohol had the same correlations with the climatic parameters (Figure 5A). In terms of the flavonoids, the concentration of anthocyanins had strong positive correlations with the MicroPAR (Figure 5B). Some previous studies investigated the impacts of light on the anthocyanins and obtained similar results, which are in agreement with the present outcomes. Several studies indicated that light exposure is necessary to the anthocyanin. [19,37,44,47]. The results also confirmed that the total flavanols concentrations in the berry skins were not correlated with the light exposure (Figure 5B). However, the concentration of (-)-epicatechin and (-)-epicatechin-3-*O*-galate in the berry skins had strong negative correlations with MicroT. The SoilW did not correlates with the marker flavonoids, except for the flavanols in the berry seeds. To be specific, (-)-epicatechin and (-)-epicatechin-3-*O*-galate had strong positive correlations with SoilW. The climatic parameters of DH20 did not correlate with all the marker aroma compounds and the marker flavonoids, which had the same outcomes with previous study [19].

### 3.6. Influence on the Chemical Parameters and the Aroma Compounds in Wines

As shown in Table 1, in the must, covering peanuts decreased the pH value and increased the titratable acidity (TA), but the effect was not significant in 2019. Regarding the wine chemical parameters, covering peanuts significantly decreased the pH value, the titratable acidity (TA) and the alcohol levels in the wine compared to CK in the two years of 2018 and 2019. There were no significant differences in the residual sugar and the volatile acidity between treatments.

**Figure 6:**
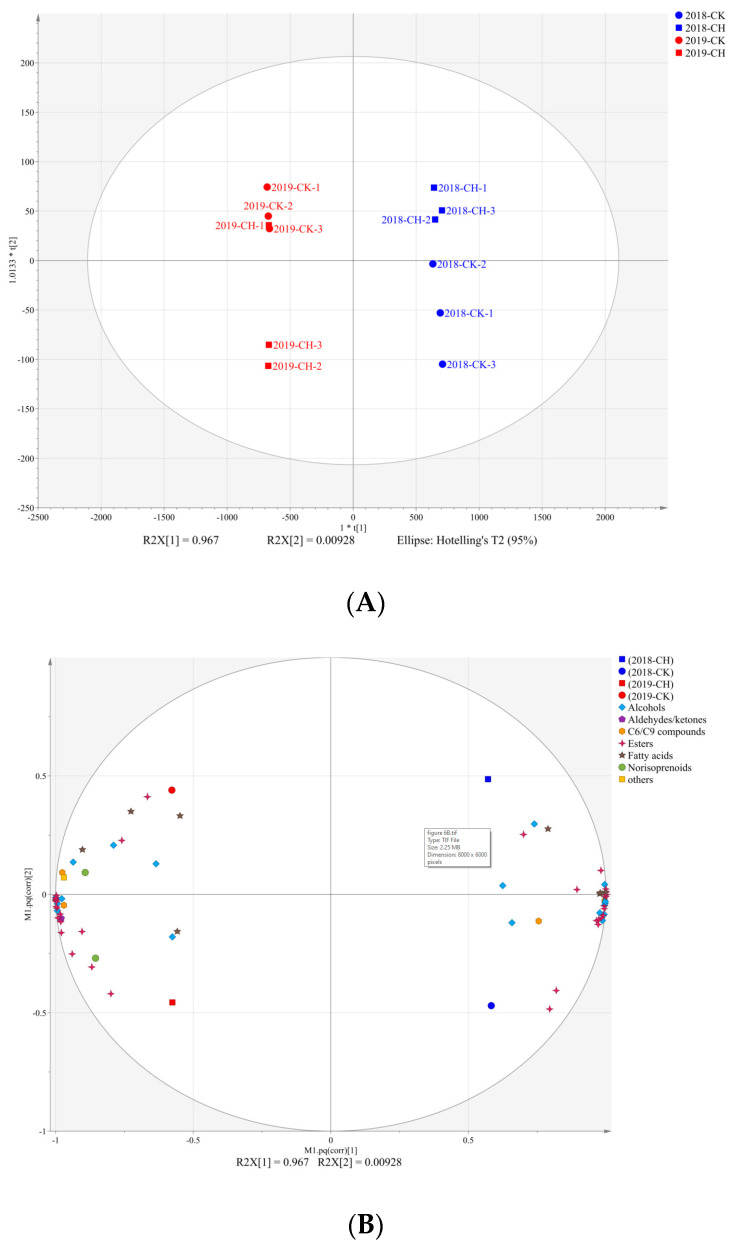
OPLS-DA analysis based on the aroma compounds in the wines of CK and CH in 2018 and 2019. (**A**) score scatter plot, (**B**) correlation loading plot.

As shown in Table S7, there were seventy-two volatile compounds in the wines. The two vintages from each other were clearly distinguished by OPLS-DA analysis (R2X [1] = 96.7%), as shown in Figure 6. The treatments of clean tillage and covering peanuts were separated by R2X [2] (R2X [2] = 0.9%). That the concentration of the marker aroma compounds was lower than the corresponding aroma detection thresholds indicates that covering peanuts had little difference in the wine sensory of the two vintages when comparing the treatments of clean tillage and covering peanuts. On the other hand, though the differences in the wine aroma compounds were limited, there were higher alcohol levels by clean tillage which might have amplified the differences on wine sensory in 2018 and 2019. Several studies have reported that covering crops affect the concentration of the aroma compounds [11,13,19,48], and the authors speculate the reason might be the water competition between the crops and the grapevines.

### 3.7. Influence on the Wine Flavonoids and the Colorimetric Parameters

**Figure 7:**
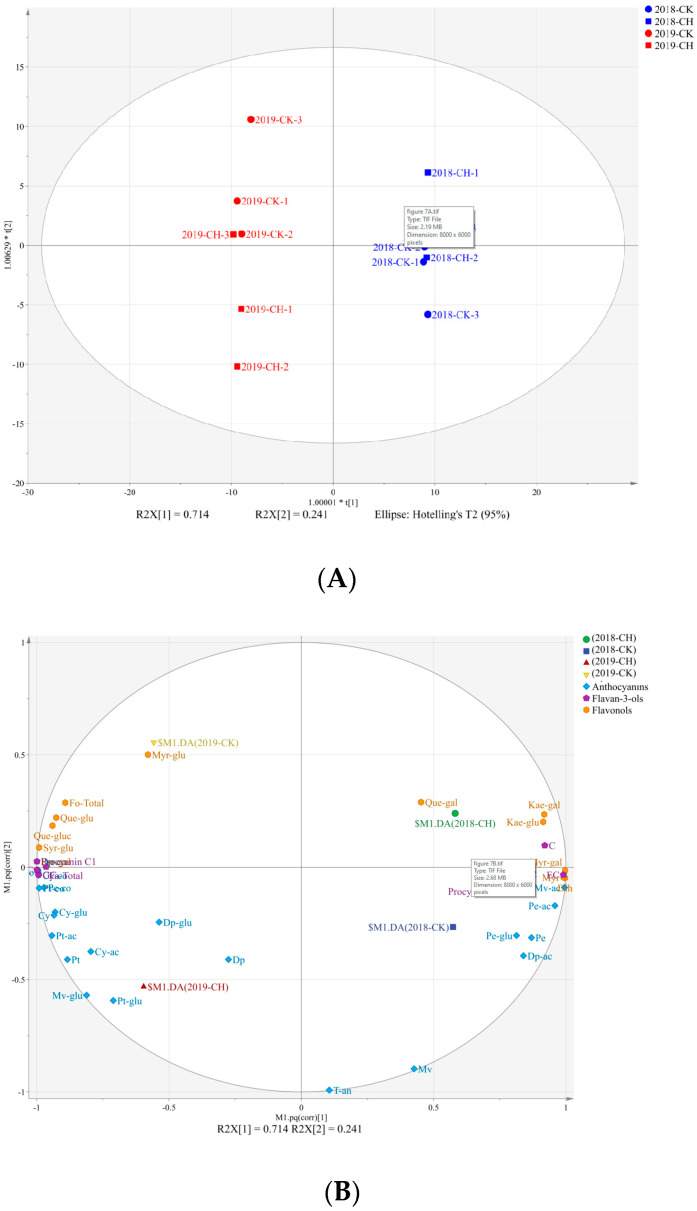
OPLS-DA based on volatile and flavonoids compounds concentrations separating wine samples according to vintage and treatment. (**A**) score scatter plot, (**B**) correlation loading plot.

In terms of the result wines, we applied the OPLS-DA analysis to investigate the effects of the treatments and years on the concentrations of the flavonoids [37]. The R2X [1] (R2X [1] = 71.4%) separated the vintages clearly (Figure 7). As shown in Figure 7 and Table S8, compared to 2018, the loading plot showed higher concentrations of the total anthocyanins, flavonols and flavan-3-ols in 2019, which was consistent with grape berries, as described above. R2X [2] explained 24.1% of the total variance and separated the clean tillage and covering peanuts. R2X [2] was positively correlated with all of the flavonols, except for myricetin-3-*O*-galactoside and myricetin, while it was negatively correlated with anthocyanins (Figure 7B). Notably, concentrations of Kaempferol-3-*O*-glucoside (Kae-glu), Quercetin-3-*O*-glucoside (Que-glu), Myricetin-3-*O*-glucoside (Myr-glu) and the total flavonols were consistently lower by covering peanuts. In addition, the concentrations of procyanidin B1 and Delphinidin-3-O-glucoside (Dp-glu) were consistently higher in the covering peanuts wines than in the CK wines (Table S8).

The concentrations of flavonols in the wines were significantly correlated with those in grapes berries. There were the same effects on the concentration of total flavonols between grape berries and wines by covering peanuts, which indicates that the effects of the treatment of covering peanuts on the flavonols in the grape berries could be reflected in the final wines. During the alcoholic fermentation, the berries skins and seeds extracted the flavan-3-ols to wines by maceration, and compared to the acid-cleaved extraction of the proanthocyanidins from the grape skins and seeds, maceration was a relatively mild extraction process [37]. Therefore, the authors speculate that the different extractabilities leading to the higher concentrations of flavan-3-ols and procyanidin B1 in the covering peanuts wines than those in the CK wines.

In 2018 and 2019, we studied the differences in the wine colorimetric parameters between clean tillage and covering peanuts, as shown in Table S7. There were higher a*, b*, C*, and lower *L* in the wines in 2019 than those of 2018. The authors speculate that lower temperature (vintage 2018) could decreased the yellow colour (b*) and colour vividness (C*) and increased the light (*L*), and this result was consistent with Peng et al. [19]. Notably, the covering peanuts wines presented lower, a*, b* and hab values than the clean tillage wines in the two years, and in 2018, the difference between the two treatments was significant, indicating that covering peanuts decreased the red colour (a*) and yellow colour (b*) of the wines (Table S8). The authors speculate that another reason was the difference in wine physicochemical parameters between vintages and between treatments. The wine pH value affects the colour expressions of anthocyanin in wine. The flavylium ions and the non-coloured carbinol bases where anthocyanins exist form in red wines. With the increase of the pH value in wines, the flavylium ions and the non-coloured carbinol bases could be converted into cis/trans-chalcones exhibiting light yellow colour [37,49,50].

**Figure 8:**
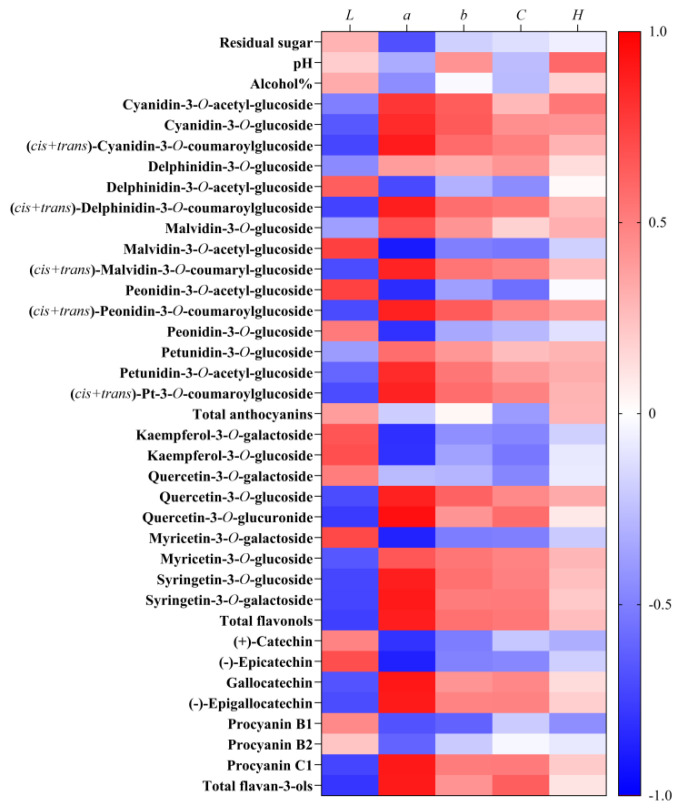
Pearson correlation analysis revealing the relationships between the wine colorimetric parameters and the residual sugar, pH value, alcohol level and the flavonoids.

In order to explain the wine colour differences either between the vintages or between the treatments, based on the wines’ colorimetric, the physiochemical parameters and the flavonoids concentrations, we conducted the correlation analysis, and the results are shown in Figure 8. The residual sugar content, pH and alcohols were positively correlated with the *L* value while negatively correlated with the a* value (Figure 8). This result is inconsistent with those of Wang Yu et al. [37]. Conversely, anthocyanins, including Cyanidin-3-*O*-acetyl-glucoside (Cy-ac), Cyanidin-3-*O*-glucoside (Cy-glu), Cyanidin-3-*O*-coumaroylglucoside (Cy-co), Delphinidin-3-*O*-glucoside (Dp-glu), Delphinidin-3-*O*-coumaroylglucoside (Dp-co), Malvidin-3-*O*-glucoside (Mv-glu), Malvidin-3-*O*-coumaryl-glucoside (Mv-co), Peonidin-3-*O*-coumaroylglucoside (Pe-co), Petunidin-3-*O*-glucoside (Pt-glu), Petunidin-3-*O*-acetyl-glucoside (Pt-ac), Petunidin-3-*O*-coumaroylglucoside (Pt-co), flavonols including Quercetin-3-*O*-glucoside (Que-glu), Quercetin-3-O-glucuronide (Que-gluc), Myricetin-3-*O*-glucoside (Myr-glu), Syringetin-3-*O*-glucoside (Syr-glu), Syringetin-3-*O*-galactoside (Syr-gal), and flavan-3-ols including Gallocatechin (GC), (-)-Epigallocatechin (EGC) and Procyanin C1 were negatively correlated with L*, while positively correlated with a*, b* C* (Figure 8).

### 3.8. Influence on the Sensory Evaluation

Sensory evaluation can intuitively show the quality of wine [51,52]. There are three main parts in wine sensory analysis, including the visual perceptions, the olfactory sensations, and the taste [53,54,55]. The senses of olfactory, taste and mouth feel are related to the specific chemical composition of wine. In terms of olfactory, acetic acid is vinegary, and formic acid has a strong pungent odour; acids are important compounds in wines. Some chemical factors in wines could affect the taste and mouth feel sensations, which further affect the wine sensory quality. The aroma compounds combined with ethanol and glycerol are associated with the sweetness of dry wines. In addition, the ethanol could balance the sour taste. In addition, the high alcohol levels could result in the roughness and hotness in wines, and further change the wine sensory profile [56]. In red wine, tannins are the typical compounds, which induced bitter and astringent sensations [57], and astringency described as woody, rough. And rough is one of the most important sensory characteristics in red wine [58,59,60,61]. Several studies reported that wine sensory analysis was the most direct method to evaluate the wine astringency [62,63].

**Figure 9:**
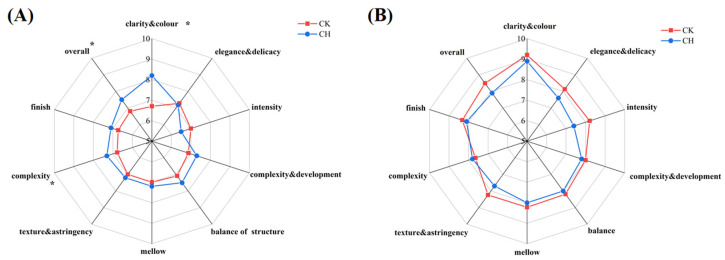
Effects of covering peanuts on the sensory evaluation of ‘Cabernet Sauvignon’ wines. (**A**) the wines of 2018; (**B**) the wines of 2019. * indicates significant differences between the control and covering purslane (*p* < 0.05, *t*-test).

As shown in Figure 9, in terms of the wine sensory, we investigated ten aspects of wines, including colour and delicacy, elegance, intensity, complexity and development, balance of structure, mellow, texture, complexity, finish, and overall. Except for the intensity, covering peanuts increased the indicators’ scores of the wines in 2018. However, in 2019, covering peanuts did not significantly affect the various wines indicators. The content of the aroma compounds and flavonoids compounds in the grape berries and wines had some correlation with this result.

In 2018, compared to clean tillage, the covering peanuts had higher score of complexity, overall, and clarity and colour. In 2019, covering peanuts did not have significant effect on the wine sensory quality. Therefore, the treatment of covering peanuts had a certain positive effect on the sensory quality improvement of the ‘Cabernet Sauvignon’ wines, as shown in Figure 9.

## 4. Conclusions

The present study analysed the effects on grape berries and wine quality by covering peanuts in the vineyard in Northwest China, including the composition of flavonoids and aroma compounds, using ‘Cabernet Sauvignon’ (*Vitis vinifera* L. cv.) as experimental material for two consecutive years of 2018 and 2019. The mesoclimate of the experimental vineyard and the microclimate around the fruit zone were monitored. The two years had the same growing season temperature, but there was higher GDD in 2019. In addition, the period after veraison in 2018 was characterized by the high duration of low (>10~15 °C) and shorter temperature (>30 °C) and the period after veraison was characterized as cooler in 2018. Covering peanuts decreased the photosynthetically active radiation and extended the duration of the low temperature (10~20 °C). As a result, covering peanuts significantly increased the total concentration of the norisoprenoids and the C_6_/C_9_ compounds in the two consecutive vintages. And covering peanuts could significantly improve the total sensory qualities of the wine, especially in the relatively cool years. The light exposure and the duration of the low and high temperature had strong correlations with the total norisoprenoids. In the semi-arid climate of Northwest China, covering peanuts decreased the photosynthetically active radiation and the high temperature duration, which resulted in decreases of anthocyanins concentrations in the grape berries, and significant decreases of the total proanthocyanidins concentrations in the grape seeds. Regarding wine flavonoids, lower concentrations of the total flavonols were consistently observed in the covering peanuts wines than in the clean tillage wines in 2018 and 2019, which resulted in the CH wines showing less light (L*), more red colour (a*) and yellow colour (b*). Even though covering peanuts increased the flavanols concentrations in the wines, there were not always consistent differences in the colorimetric parameters between the clean tillage and the covering peanuts wines in the two vintages.

The present study analysed the effects on grapes and wine quality by covering peanuts in the vineyard in Northwest China, including the composition of flavonoids and aroma compounds, using ‘Cabernet Sauvignon’ (Vitis vinifera L. cv.) as experimental material for two consecutive years in 2018 and 2019. The results highlight the importance of the solar radiation and the concurrent microclimate changes to the accumulation of the aroma compounds and the flavonoids in grapes and their resulting wines’ aroma and color expression by using peanuts growing between rows. In a practical sense, peanuts are an ideal plant that could regulate the microclimate and have extra economic value in that region, whereas covering peanuts had limited influence on the anthocyanins and the proanthocyanidins accumulation, as well as their expression in the resulting wines to produce premium products there.

## Figures and Tables

**Table 1:** Must and wine physicochemical parameters of the clean tillage (CK) and covering peanuts (CH).

Fermentation Stage	Years	Parameter	Treatment		Significance
			CK	CH	
Must	2018	TSS (°Brix)	23.47 ± 0.18	24.03 ± 0.21	Ns
		TA (g/L)	8.90 ± 0.13	9.83 ± 0.22	*
		pH	3.48 ± 0.01	3.42 ± 0.02	*
	2019	TSS (°Brix)	24.03 ± 0.22	23.03 ± 0.06	*
		TA (g/L)	7.34 ± 0.23	7.91 ± 0.22	Ns
		pH	3.42 ± 0.01	3.39 ± 0.01	Ns
Wine	2018	Residual sugar (g/L)	1.98 ± 0.33	2.05 ± 0.05	Ns
		pH	4.09 ± 0.01	4.05 ± 0.02	*
		TA (g/L)	4.95 ± 0.19	5.91 ± 0.07	*
		Alcohol degree (%, *v*/*v*)	12.90 ± 0.58	12.53 ± 0.06	*
		Volatile acidity (g/L)	0.51 ± 0.03	0.53 ± 0.05	Ns
	2019	Residual sugar (g/L)	1.73 ± 0.11	1.35 ± 0.44	Ns
		pH	4.07 ± 0.12	4.02 ± 0.02	*
		TA (g/L)	4.90 ± 0.01	5.87 ± 0.10	*
		Alcohol degree (%, *v*/*v*)	12.57 ± 0.07	12.03 ± 0.06	*
		Volatile acidity (g/L)	0.55 ± 0.02	0.50 ± 0.03	Ns

Values are reported as means ± SD of three biological replicates, * indicates significant differences between the control and covering purslane (*p* < 0.05, *t*-test). NS = not significant.

## Data Availability

Data is contained within the article or supplementary material.

## Supplementary Materials

The following supporting information can be downloaded at: https://www.mdpi.com/article/10.3390/foods11223730/s1, Table S1: Bioclimatic indices of three vintages (2018–2019); Table S2: Phenological data and climatic parameters of the vineyard in 2018–2019; Table S3: Average daily water content (SoilW); fruit-zone average daily temperature (MicroT), average daily photosynthetically active radiation (MicroPAR), and degree hour indexes within different temperature intervals (DH10, 10–15 °C; DH20, 20–25 °C; DH25, 25–30 °C; DH30, >30 °C) of the period from veraison to harvest in the year of 2018 and 2019; Table S4: Vine parameters of clean tillage control (CK) and covering peanuts (CH) of ‘Cabernet-Sauvignon’ in the year of 2019; Table S5: Aroma compounds concentrations in mature grapes in the year of 2018 and 2019 (mg/kg berry fresh weight); Table S6: Flavonoids concentrations and proportions in mature grapes in the year of 2018 and 2019; Table S7: Volatile compounds concentrations in wines in the year of 2018 and 2019 (μg/L); Table S8: Flavonoid concentrations (μg/L) in wines in 2018–2019.
